# Supplementary material for: Glycans are not necessary to maintain the pathobiological features of bovine spongiform encephalopathy
Source: PLoS Pathog. 2022 Oct 7;18(10):e1010900. doi: 10.1371/journal.ppat.1010900 (PMC9581369; doi:10.1371/journal.ppat.1010900)
Supplement: S1 Fig — Samples were digested with 170 μg/mL PK and 3 ng/μL PNGase F, loaded on SDS-PAGE and subjected to Western Blot using monoclonal antibody 3F4 (1:10,000). Note that PNGase treatment reduced vCJD-infected Tg340 three-band pattern to a single band of approximately 19 kDa, but did not affect the banding pattern of vCJD-PMCA-infected TgNN6h, which in addition retained the ~15-kDa band that we have previously observed in this unglycosylated model, assumed to be an endogenous proteolytic fragment. PNGase digestion did not cause the emergence of the 15-kDa band in Tg340 samples, indicating that it is not the unglycosylated form of a fragment present in Tg340 brains. (DOCX) [file ppat.1010900.s001.docx]

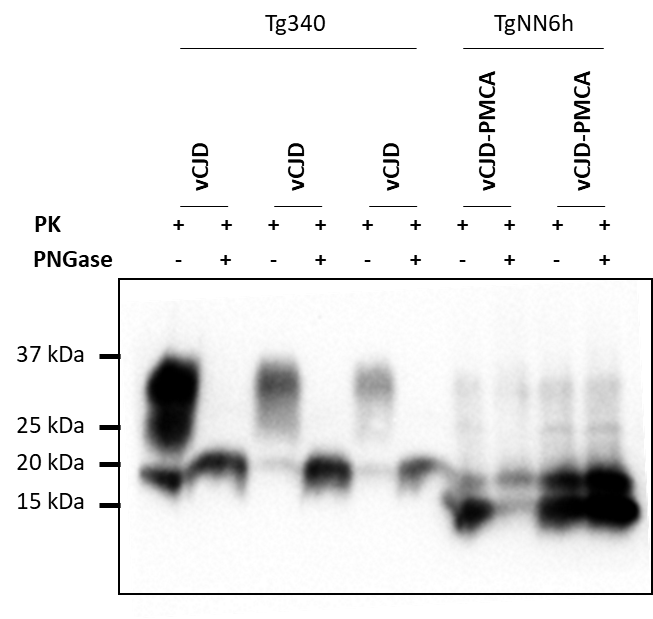


**S1 Fig. PNGase digestion of vCJD-infected Tg340 and vCJD-PMCA-TgNN6h mice brains.** Samples were digested with 170 μg/mL PK and 3 ng/μL PNGase F, loaded on SDS-PAGE and subjected to Western Blot using monoclonal antibody 3F4 (1:10,000). Note that PNGase treatment reduced vCJD-infected Tg340 three-band pattern to a single band of approximately 19 kDa, but did not affect the banding pattern of vCJD-PMCA-infected TgNN6h, which in addition retained the ~15-kDa band that we have previously observed in this unglycosylated model, assumed to be an endogenous proteolytic fragment. PNGase digestion did not cause the emergence of the 15-kDa band in Tg340 samples, indicating that it is not the unglycosylated form of a fragment present in Tg340 brains.
