# Supplementary material for: Glycans are not necessary to maintain the pathobiological features of bovine spongiform encephalopathy
Source: PLoS Pathog. 2022 Oct 7;18(10):e1010900. doi: 10.1371/journal.ppat.1010900 (PMC9581369; doi:10.1371/journal.ppat.1010900)
Supplement: S2 Fig — The core and the radiating spicules of the florid plaques observed in TgNN6h mice were Congo Red positive, indicating an amyloid fibrils organization. (DOCX) [file ppat.1010900.s002.docx]

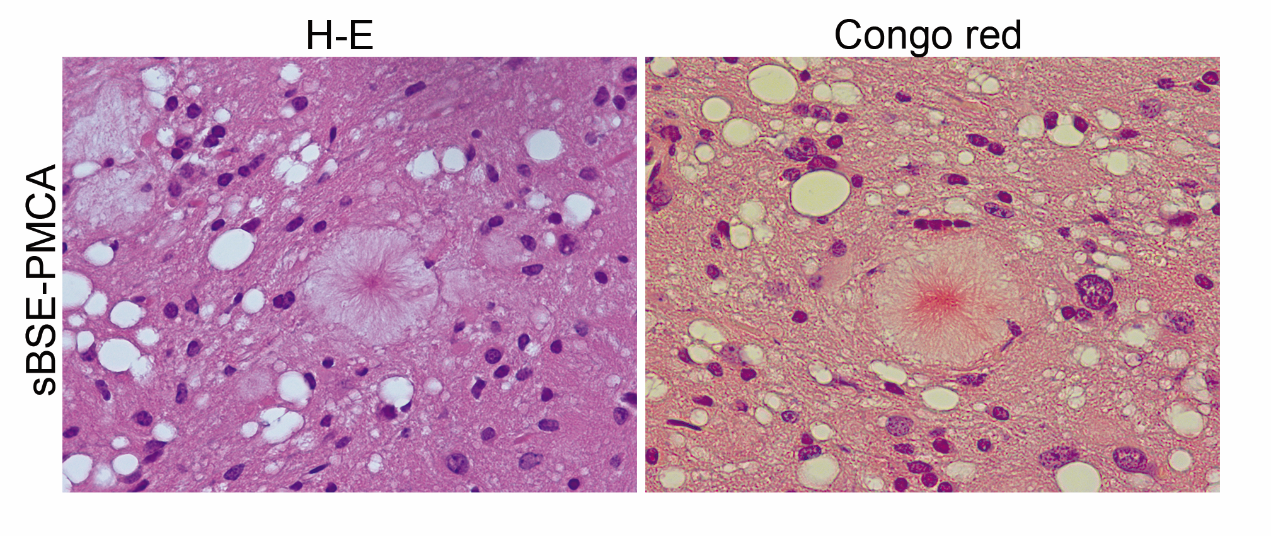


**S2 Fig. Hematoxylin-eosin and Congo Red staining of a florid plaque observed in the thalamus of a sBSE-PMCA inoculated TgNN6h mouse (x40).** The core and the radiating spicules of the florid plaques observed in TgNN6h mice were Congo Red positive, indicating an amyloid fibrils organization.
