# Supplementary figures and images for: Glycans are not necessary to maintain the pathobiological features of bovine spongiform encephalopathy
Source: PLoS Pathog. 2022 Oct 7;18(10):e1010900. doi: 10.1371/journal.ppat.1010900 (PMC9581369; doi:10.1371/journal.ppat.1010900)

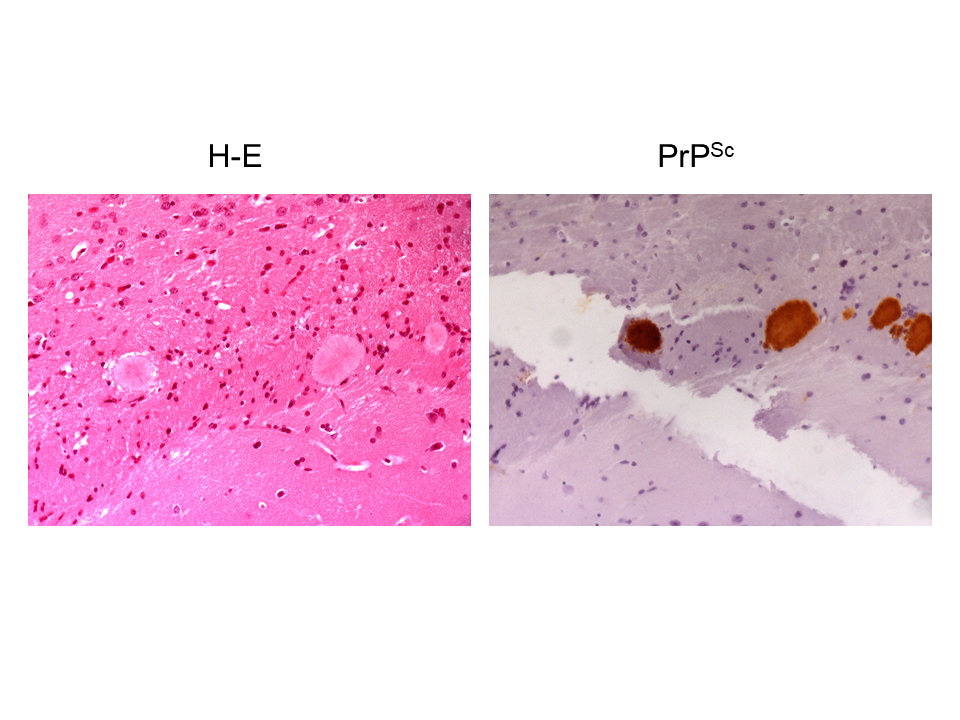


**S3 Fig. Florid plaques observed in the subcallosal area of a Tg340 mouse inoculated with vCJD.**

Supplement: S3 Fig — Florid plaques observed in the subcallosal area of a Tg340 mouse inoculated with vCJD. (DOCX) [file ppat.1010900.s003.docx]
