# Supplementary material for: Glycans are not necessary to maintain the pathobiological features of bovine spongiform encephalopathy
Source: PLoS Pathog. 2022 Oct 7;18(10):e1010900. doi: 10.1371/journal.ppat.1010900 (PMC9581369; doi:10.1371/journal.ppat.1010900)
Supplement: S4 Fig — Two serial PMCA rounds of these inocula were performed on BoTg110 substrate, and the PMCA products were digested with 85 μg/mL PK, loaded on SDS-PAGE and subjected to Western Blot using monoclonal antibody 6H4 (1:10,000); only the second PMCA round is shown. Note that, while the original inoculum (from cattle) was able to propagate down to a 10−5 dilution in two rounds of serial PMCA, the TgNN6h-passaged inocula were able to amplify only at a 10−1 dilution, indicating that their infectious titers were significant lower. (DOCX) [file ppat.1010900.s004.docx]

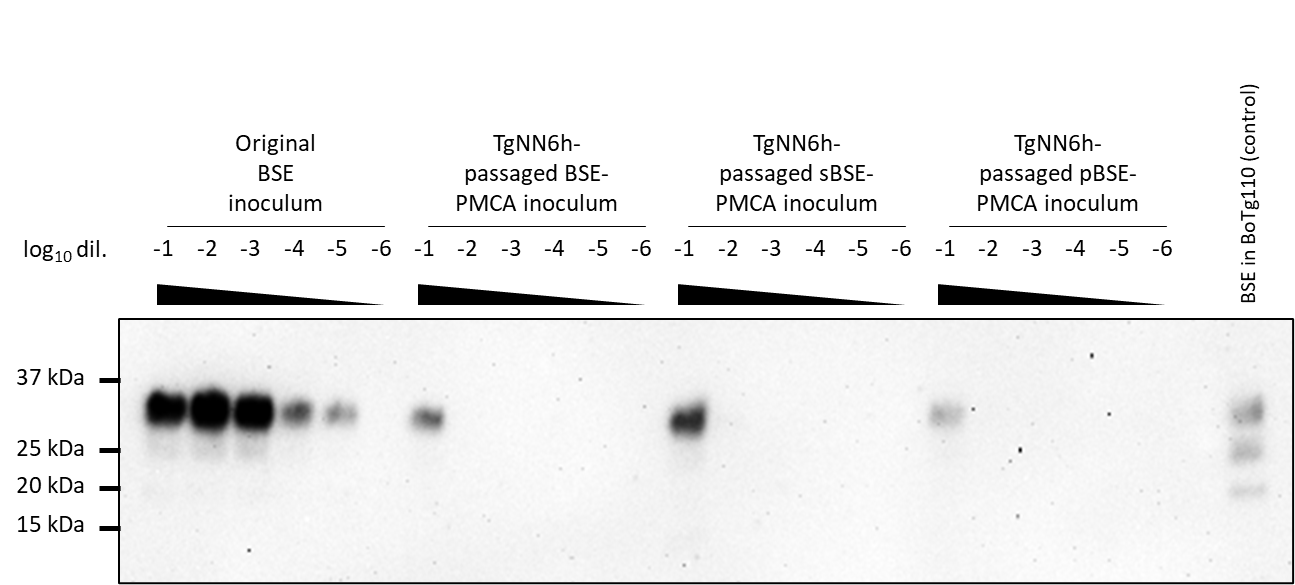


**S4 Fig. *In vitro* titration of BSE infectivity present in the original BSE inoculum and the PMCA-adapted, then TgNN6h-passaged BSE, sBSE and pBSE inocula**. Two serial PMCA rounds of these inocula were performed on BoTg110 substrate, and the PMCA products were digested with 85 μg/mL PK, loaded on SDS-PAGE and subjected to Western Blot using monoclonal antibody 6H4 (1:10,000); only the second PMCA round is shown.  Note that, while the original inoculum (from cattle) was able to propagate down to a 10^-5^ dilution in two rounds of serial PMCA, the TgNN6h-passaged inocula were able to amplify only at a 10^-1^ dilution, indicating that their infectious titers were significant lower.
