# Supplementary material for: Glycans are not necessary to maintain the pathobiological features of bovine spongiform encephalopathy
Source: PLoS Pathog. 2022 Oct 7;18(10):e1010900. doi: 10.1371/journal.ppat.1010900 (PMC9581369; doi:10.1371/journal.ppat.1010900)
Supplement: Attachment — Submitted filename: Otero et al. Response to reviewers.docx [file ppat.1010900.s005.docx]

Derio, August 29^th^, 2022

Dear editor,

Re: Manuscript reference PPATHOGENS-D-22-00771

Thank you very much for inviting us to submit a reviewed version of our manuscript "**Glycans are not necessary to maintain the pathobiological features of bovine spongiform encephalopathy**". We also thank the reviewers for their time and their constructive and insightful comments, which undoubtedly have helped us to improve its quality. We have addressed each of their concerns providing a point-by-point response to the observations and questions about the manuscript as described below.

As suggested, answers to the concerns of the reviewers’ comments are indicated in blue color font. We have also included a new supplementary figure with their respective footnotes and in-text mentions in order to improve the manuscript, answer the reviewers’ concerns and strengthen our conclusions.

**List of points raised by the reviewers:**

**Reviewer #1:** The normal form of the prion protein PrPC can be variably occupied by glycans at two asparagine amino acids located in the C-terminal core. In the pathological PrPSc form, there are stoichiometric ratios of glycoforms attached to the protein. Whether PrPSc glycosylation is required in maintaining prion strain properties and in facilitating prion transmission within and between species has been an ongoing, fascinating debate for the last 20 years. Any insight on these topics will help further understanding the biology of these agents, including during adaptative events. In this context, Otero and collaborators report on the absence of glycosylation requirements to maintain the strain properties of BSE prions on adaptation to human PrP sequence.

Methodologically, the authors built on transgenic mice expressing unglycosylated human PrP (the previously published tgNN6h mouse line) that was used for bioassay or for brain substrate for in-house PMCA experiments. BSE prions were from cattle or passaged through different farmed species or human under the form of vCJD.

Overall, the study is well constructed, the results are straightforward and manuscript is very clear.

I have several questions and minor comments.

*Major:*

- In table 1, the authors show that the different BSE/vCJD-PMCA samples readily propagate in tgNN6h mice. Did the authors inoculate tgNN6h mice with the same BSE/vCJD prions amplified by PMCA onto tg340 mice expressing glycosylated PrPC to further examine if the absence of glycans facilitate the propagation of these agents? This is suggested by the observation that vCJD prions propagate at similar attack rate and incubation periods in tgNN6h compared to tg340 mice despite 6-fold decreased PrPC expression levels.

Although it is true that TgNN6h readily propagated PMCA-adapted BSE/vCJD inocula, likely due to PrPC sequence identity between inoculum and host, we do not completely agree with the reviewer in that non PMCA-adapted vCJD prions propagated similarly in TgNN6h and Tg340 mice. The former showed an attack rate of 6 out of 11 (54%), while the latter succumbed with an attack rate of 4 out of 5 (80%). In addition, the mean incubation time for Tg340 was 81 days shorter (a 10% difference) than that of TgNN6h. In our opinion, the 6-fold lower expression levels of the TgNN6h model could sufficiently explain such difference, and thus, would not indicate a facilitated transmission in unglycosylated substrate. Although the experiment proposed would be interesting, in light of the results obtained from the direct inoculation of fully glycosylated vCJD in both models, we believe that adaptation of BSE inocula to Tg340 mice and subsequent inoculation in TgNN6h mice would likely result negative. The attack rates and incubation times were worse in the case of vCJD in TgNN6h mice than were in Tg340, and the other inocula were negative in Tg340 mice (with the exception of pBSE, with only one animal succumbing to disease). Therefore, we consider that the conclusions that could be withdrawn do not merit such a long and laborious experiment, although we appreciate the reviewer’s suggestion.

- Did the authors inoculated the BSE/vCJD-PMCA products to tg340 mice to further sustain the view that the strains properties were maintained despite amplification on a deglycosylated PrPC substrate?

Although we agree with the reviewer that exploring further the properties of unglycosylated BSE prions by bioassay in humanized mice would have been interesting, the focus of this work was to assess whether BSE would maintain its hallmarks in an unglycosylated model. Therefore, we consider that using a model engineered to express the PrPC of the BSE original species (cattle) is most appropriate to assess whether BSE goes back to its original form. Trying to address this issue using Tg340 instead would have made our conclusions more hazy, as well as our experiments longer since this model does not propagate this strain as efficiently as the BoTg110 model.

We have now included a paragraph in the discussion (lanes 431-436) stating the interest of performing this alternative experiment:

In addition to this, the back-passage of these unglycosylated prions in their original PrP^C^ environment, i.e. BoTg110 mice expressing wild-type cattle PrP^C^, resulted in the recovery of the BSE full phenotype. BoTg110 mice were chosen for this study as they transmit the BSE agent in absence of any transmission barrier, and are thus the most suitable model to assess whether unglycosylated BSE prions recover their prototypal characteristics. Alternatively, a back-passage of the TgNN6h-propagated BSE isolates into humanized, instead of bovinized, transgenic mice would also provide interesting clues on the properties of these unglycosylated BSE prions. Overall, thus, our results suggest that glycans are not vital in the determination of this transmission barrier or for the conservation of the pathobiological features of BSE prions.

- The authors found that PrPSc found in the brains of tgNNH6 mice inoculated with BSE/vCJD-PMCA samples exhibit a vCJD-like electrophoretic signature, with unglycosylated PrPSc migrating at 19-20 kDa. They also found an additional band at 15 kDa. Is this band also observed in tg340 mice inoculated with vCJD/BSE samples after PNGase deglycosylation?

As suggested by the reviewer, samples from Tg340 mice inoculated with vCJD and BSE were analyzed after deglycosylation to assess whether the 15 kDa band observed in TgNN6h mice could also be found in these animals. We have now included a Supplementary figure (Supplementary figure 1 and the corresponding description of the procedure in Materials and Methods section) showing the same samples (three Tg340 mouse brains inoculated with vCJD and two TgNN6h mouse brains also inoculated with vCJD, previously adapted to TgNN6h brain in PMCA) untreated and treated with PNGase F after PK digestion. As shown in the figure, deglycosylated Tg340 mice showed only a 19-20 kDa band, in contrast to the deglycosylated TgNN6h mice, which still showed the 15 kDa band. Although we did not further characterize this 15 kDa band from BSE-infected TgNN6h mice, given that it is also present in undigested samples from uninfected TgNN6h mice, we assume that it is an endogenous proteolytic fragment or truncated form similar to those described in other models (Lewis, Johanssen et al. 2016, ref. number 64). Since histopathological analysis of these animals clearly suggests conservation of strain properties of BSE, we consider that this fragment is just a particular feature of this animal model.

A sentence referring to this new Supplementary figure was included in the Results section (Lanes 199-203):

A lower band (around 15 kDa) was consistently observed only in these animals (Figure 3), but not in Tg340 mice. Deglycosylation of BSE-infected TgNN6h samples with PNGase F did not provoke a shift of this band (Supplementary figure 1). In addition, Tg340 samples treated with PNGase F showed a reduction of the three-bands pattern to a single 19-20 kDa band, but it did not show the 15 kDa band (Supplementary figure 1). Therefore, we believe that this band corresponds to an endoproteolytic fragment specific to TgNN6h mice, although the reason why it only arises from unglycosylated PrP^Sc^ remains to be investigated.

Also, a comment has been included in Discussion section (Lanes 370-375):

This lower fragment can be also observed in undigested TgNN6h substrate (Figure 2), but not in Tg340 mice inoculated with vCJD, not even after deglycosylation (Suplementary figure 1). Although we did not further characterized this 15 kDa band seemingly characteristic of TgNN6h mice, we believe that it may correspond to an endoproteolytic fragment similar to others previously described (64), and that apparently occur preferentially on unglycosylated substrate.

And a paragraph has been introduced in the Material and Methods section explaining the technical details of this new experiment (lanes 529-535):

PNGase treatment was performed in 10 % brain homogenates from vCJD inoculated Tg340 and TgNN6h animals after PK digestion, using CarboClip PNGase F (Asparia Glycomics) and following instructions from the supplier. Briefly, the denaturing buffer (5 % SDS, 400 mM DTT) was added to previously digested brain homogenates at 1:10 dilution and they were incubated at 100 °C for 10 min. After adding the reaction buffer (5 mM sodium phosphate, pH 7.5 with 1 % NP-40) and PNGase F at 3 ng/μl, samples were incubated for 1 h at 37 °C. Finally 4x NuPAGE LDS buffer was added and samples analyzed by Western blot as described above.

- It is a bit surprising that BSE/vCJD-PMCA passaged twice on tgNN6h reinfected parental bovine PrP mice at incomplete attack rate and longer incubation time than cattle BSE. As the PrPSc signature found in the brain of the sick mice is similar to that of BSE in the same model, the authors conclude that the strain properties of BSE have been recovered. Strictly speaking, a second passage would be necessary for a more definitive conclusion. To explain this apparent discrepancy, the authors suggest that the infectivity levels of BSE/vCJD prions in tgNN6h mice may be lowered, due to the low levels of PrPC expression in these mice. At least two articles contradict this hypothesis: 1) heterozygous mice harbour similar infectivity levels than wild-type mice at terminal stage, despite extended incubation time (Bueler, et al., 1994); 2) scrapie prions infectivity levels are similar between wt animals and overexpressing mice (Douet, et al., 2014). To address this issue, the authors could titrate the seeding activity of their brains compared to cattle BSE brain.

We agree with the reviewer that the incomplete attack rates in BoTg110 mice are unexpected when, seemingly, strain features were conserved upon passage in the unglycosylated model and that a second passage would definitely address this issue. Nonetheless, since biochemical and histopathological features of BoTg110 mice that succumbed to the disease showed clearly a conservation of BSE strain features, we deemed unnecessary to perform such long and animal costly experiment to fulfill our goal of demonstrating that BSE strain features are not altered in absence of glycosylation. To provide an explanation of these incomplete attack rates, we theorized the possibility of lower expression levels in TgNN6 being responsible for such observation although we have to admit that the reviewer is right in pointing that there is evidence against this explanation. As suggested, we performed an *in vitro* titration experiment to try to solve this issue, comparing the propagation of the cattle BSE isolate with that of the BSE, sBSE, and pBSE-inoculated TgNN6h brain homogenates that were used to inoculate BoTg110 mice. As explained in the now revised version of the Materials and Methods section, the four brain homogenates were serially diluted (10^-1^ to 10^-6^) and used as seeds on PMCA using a substrate prepared from healthy BoTg110 mice. All the samples were subjected to two serial 24 h rounds of PMCA in the same conditions as previous PMCA reactions, and performing a 1:10 dilution in between the two serial rounds. PMCA products from the second round were analyzed by Western blot, which is shown now as Supplementary figure 4. As shown in the gel, the original cattle BSE isolate was detectable up to dilution 10^-5^, whereas the three TgNN6h brain homogenates were only able to propagate up to dilution 10^-1^, i.e. a 10,000-fold lower *in vitro* propagation capability. This lower titer, together with the prolonged incubation times characteristic of BSE isolates, could explain the incomplete attack rates observed. Alternative explanations for the incomplete attack rate are also possible and they are presented in the discussion, notably the existence of a transmission barrier derived from the PrP amino acid sequence differences between TgNN6h and BoTg110 mice, which despite conservation of strain properties could have prolonged incubation times in BoTg110 mice over the lifespan of these animal models. Again, a second passage in BoTg110 animals would definitely provide an answer to this question. However, we considered it unnecessary in light of the biochemical and histopathological data that allows firmly concluding a conservation of the highly characteristic features of BSE and derived isolates.

The Results section now includes a paragraph explaining the results of this new experiment (lanes 242-262):

While cattle BSE readily transmitted to BoTg110 mice (100% attack rate) and incubation periods of around 300 dpi, as previously reported ([4](https://docs.google.com/document/d/1MZnjJjTBlrNTeELYaGgOCkbKZHVikUSe/edit#heading=h.2jxsxqh), [7](https://docs.google.com/document/d/1MZnjJjTBlrNTeELYaGgOCkbKZHVikUSe/edit#heading=h.1y810tw)), all three non-glycosylated isolates propagated with incomplete attack rates and longer and heterogeneous incubation periods (Table 2). These discrepant results are unlikely to reflect a change in the strain properties of BSE prions upon passage in the TgNN6h model (since, as stated below, the biochemical and neuropathological hallmarks were identical to those obtained with the original cattle BSE isolate). Rather, they evidence the transmission barrier naturally occurring between cattle and human, likely increased by the two additional amino acid changes introduced in the TgNN6h PrP^C^ sequence to impede glycosylation.

Two additional factors may have contributed to this reduced transmission efficiency: 1) the fact that TgNN6h mouse brains express ~40 % less PrP^C^ than a human brain, thus accumulating lower amounts of PrP^Sc^ and infectivity, and 2) the use of 1 % (instead of the standard 10 %) brain homogenates as inocula, which further reduces the infective dose administered to each animal. In order to prove this hypothesis, we performed an *in vitro* titration experiment. Both the original cattle BSE isolate and the BSE-TgNN6h, sBSE-TgNN6h, and pBSE-TgNN6h inocula that were used to infect BoTg110 mice were serially diluted and submitted to two serial PMCA rounds in BoTg110 substrate. While cattle BSE was able to propagate up to dilution 10^-5^, all of the TgNN6h-passaged isolates propagated only up to dilution 10^-1^ (Supplementary figure 4). These results allowed us to conclude that the propagative dose that accumulates in the brain of TgNN6h mice is significantly lower than that of BSE-infected cattle, thus explaining the low transmission efficiency of these isolates in first-passage BoTg110 mice.

Also, the Discussion section has been modified to contemplate all the possibilities described above (lanes 381-407):

These TgNN6h-passaged BSE prions, however, transmitted with incomplete attack rates to BoTg110 mice, in contrast with the results obtained by Padilla and colleagues with normally glycosylated BSE prions. These discrepant results could be explained by the transmission barrier existing between human and bovine PrP sequences, likely increased due to the presence of two additional asparagine-to-glutamine substitutions in the TgNN6h model to avoid glycosylation. Also, the fact that TgNN6h mouse brains contain ~40 % less PrP^C^ than human brain could lead to a lower accumulation of PrP^Sc^ and infectivity in this model, and the use of 1 % brain homogenates for inoculation, instead of the 10 % used in previous studies, could contribute to a reduction in the infective dose administered to each BoTg110 animal. A second passage in the same animal model would definitely demonstrate that the incomplete attack rates observed in first-passage BoTg110 animals are due to the existence of such transmission barrier and/or to reduced infectivity accumulating in TgNN6h brains, rather than to an alteration in the strain features of BSE upon passage in the unglycosylated model. In fact, neuropathological features in BoTg110 mice inoculated with the TgNN6h-passaged BSE prions were very similar from those reported by us and other authors in BoTg110 mice inoculated with fully glycosylated BSE prions, regarding distribution (43, 44) and morphology of PrP^Sc^ deposits (4, 43). Therefore, a second passage in BoTg110 was deemed unnecessary to prove that propagation upon an unglycosylated PrP^C^ does not alter BSE strain features. Nonetheless, in order to confirm that TgNN6h mouse brains accumulate less infectivity with respect to the classical BSE isolate from cattle, an *in vitro* titration experiment was performed by submitting serial dilutions of all the inocula to two serial PMCA rounds in BoTg110 substrate. As shown in Supplementary figure 4, original cattle BSE isolate was detectable up to dilution 10^-5^ in the second PMCA round, whereas the three inocula derived from TgNN6h brain homogenates were able to propagate only up to dilution 10^-1^, i.e. a 10,000-fold lower propagation capability. This apparently lower titer, together with a transmission barrier and the long incubation times characteristic of this strain, can explain the incomplete attack rates observed *in vivo*.

And a paragraph has been introduced in the Material and Methods section explaining the technical details of this new experiment (lanes 490-495):

For the *in vitro* titration of the original cattle BSE isolate and BSE, sBSE, and pBSE-infected TgNN6h brain homogenates that were used to inoculate BoTg110 mice, 10 % brain homogenates were serially diluted (10^-1^ to 10^-6^) and used as seeds on a substrate prepared from healthy BoTg110 mice. All the samples were subjected to two serial 24-hour rounds of PMCA in the same conditions described above, and performing 1:10 dilution in between the two serial rounds. PMCA products from the second round were analyzed as described below.

Maybe BSE/vCJD serially passaged on tg340 mice would have been a better control for back-passage into bovine PrP mice.

Providing more information or data would strengthen the view that unglycosylated BSE prions did not evolve phenotypically.

Although we agree that these experiments would be of interest, we think that BoTg110 mice, which expresses the PrP^C^ sequence of the BSE original species (cattle), is the most suitable model to investigate whether BSE prions recover their prototypal characteristics after propagation in a glycan-free environment. As mentioned above, we have however included one paragraph in the Discussion (lanes 431-436) on the interest of such an alternative experiment.

- Histopathological analyses unambiguously show the presence of florid plaques in the inoculated tgNNh6 mice. This type of plaque is archetypical of vCJD/BSE propagated on the human PrP sequence. Does this suggest that in mice expressing wild-type human PrP or even in humans, these plaques are preferentially formed from unglycosylated PrPSc? The authors may have referred to the work published by Sevillano et al. who showed that glycans reduced plaque formation in prion disease (Sevillano, et al., 2020).

This is an interesting suggestion. We have discussed this idea in the manuscript with the results obtained by Sevillano et al., 2020, in the Discussion section (lines 355-366):

Mice inoculated with BSE-PMCA, sBSE-PMCA, pBSE-PMCA and vCJD-PMCA disclosed very severe neuropathological changes, and showed abundant deposits with the characteristics of florid plaques described in vCJD-affected patients and humanized models (Figure 4) (7, 42). Unglycosylated prions have been reported to favor extracellular plaque formation in murine models of prion disease, in which these prions colocalize with plaque deposits (62). These results, together with the results obtained in the present study, suggest that florid plaques observed in humanized mice and humans are preferentially formed from unglycosilated PrP^Sc^. Our PMCA-propagated inocula were very similar with respect to the distribution of neuropathological changes (Figure 3), which also coincided with those previously described for Tg340 (7) and Tg650 (63) mice (expressing fully glycosylated human PrP^C^) inoculated with BSE and BSE-related prions. Overall, our results indicate that the neuropathological hallmarks of BSE were maintained after transmission to a humanized non-glycosylated host.

*Minor:*

- Lane 176 : the authors mention that the disease incidence in tg340 mice inoculated with BSE/vCJD isolates differ from that published by Padilla et al. (Padilla, et al., 2011). They pointed out correctly that inoculations were made at 1% in their lab compared to 10% in Padilla’s work. My understanding is also that the isolates used (cattle BSE, sheep BSE, vCJD) are different and may harbour different levels of infectivity.

Thank you for your suggestion. This comment has been included in the manuscript (Results, lines 184-186):

In addition, the BSE and vCJD isolates used were different from those inoculated in the present study, and therefore it is possible that these isolates contained different levels of infectivity. This, together with the prolonged incubation times close to the lifespan of the animals observed also in previous studies, could explain the absence of transmission in our case.

- Lane 255: I would replace “…cross-species transmission of prions to humans…” by “…the potential cross-species transmission of prions to humans…” to avoid any misinterpretation.

The statement has been modified in the manuscript according to the reviewer’s suggestion (now on lane 290).

- Lane 357: 127S and not 126S

Thanks, this error has been corrected.

**Reviewer #2:** Prion protein glycosylation can influence how prions replicate and cause disease in the host in a strain-specific manner. It can also impact cross species formation of prions. The manuscript by Otero et al. examines how glycosylation of the normal human prion protein (PrPC) effects prion formation and disease using BSE prions derived from cattle, sheep, pigs, and humans (i.e. vCJD). They find that the absence of glycosylation in human PrPC does not alter the BSE strain phenotype. They conclude that glycosylation of human PrPC is not essential for preserving either BSE strain properties or the species barrier to transmission of BSE to humans.

This is a straightforward, solid study using a mix of in vitro prion replication assays and in vivo bioassays to assess the role of PrP glycosylation on the transmission and strain properties of BSE. The data are clearly presented and appropriately interpreted and add to our understanding of how prion protein glycosylation can impact prion pathogenesis.

*Minor comments*

1) The authors should clarify the statement on line 76 that “expression of PrPC in the cellular membrane is necessary for the development pf prion disease”. Prion disease does in fact develop in mice that express PrPC without the GPI anchor (PLoS Pathog 6(3): e1000800. (2010)), which is secreted from the cell and is not anchored to the cell surface.

We agree with the reviewer’s suggestion. This statement has been modified in the introduction (lanes 75-81):

Several studies have demonstrated the key role of N-linked glycans in the intracellular trafficking and membrane location of PrP^C^ (15-19). This protein has a still-elusive function (20, 21). Although it was thought that the expression of PrP^C^ in the cellular membrane was fundamental for the development of prion disease (22-25), it has been shown that mice expressing an anchorless PrP^C^ (lacking the GPI attachment to the cell membrane) can develop a fatal transmissible amyloid encephalopathy (26).

2) In Figure 3, PrPC substrate from TgZW mice is shown on the western blot. TgZW mice are only mentioned in the Figure 3 legend and are described as expressing “normally glycosylated human PrPC”. More information on this line needs to be provided in the Methods if they are going to be used as a control. Why wasn’t substrate from Tg340 mice, which also make normally glycosylated human PrPC, used on these blots? Since those were the mice used for the in vivo bioassay, that would seem to be a more appropriate control.

The reviewer is right that no reference of the TgWV is given anywhere in the manuscript. We apologize for this omission, as well as for misspelling the name of the model (TgWV), which has been corrected in Figure 3 image and caption. We have now included a mention to this transgenic model in the Methods section, lanes 523-525:

Samples were run in parallel with either undigested TgNN6h, human, or cattle substrate, or undigested samples from healthy TgNN6h (unglycosylated human PrP^C^) or TgWV mice (normally glycosylated human PrP^C^ (69), as controls.

The reason for using an undigested sample from this line and not from a Tg340 mouse is purely practical: undigested brain homogenates from Tg340 mouse do not run well in gels, and so for the purpose of having a neat control of undigested normally glycosylated human PrP^C^, we preferred to use a control that, in our hands, works better on Western blot.

3) There is no reference to Figure 5 in the text where the data are discussed (lines 242-245) and this should be added. In addition, the red arrows in Figure 5 need to be defined in the legend. Presumably, they indicate passage history, but this is not clear.

We apologize for this omission. The changes suggested are now included in the manuscript.

4) The description of the neuropathology in the TgBo110 mice is quite brief (lines 246-248) and makes no mention of the fact that it is significantly different from that in the TgNN6h mice. PrPSc deposition is fine-punctate and plaque-like in the TgBo110 mice (Figure 6) versus florid plaques in Tgnn6h mice (Figure 4). In addition, the pattern of spongiform change is clearly different (compare top panels in Figures 6A and 4A). The neuropathology of BSE is generally similar when transmitted across multiple species so the difference in pathology between the two transgenic mouse lines is somewhat unexpected. It would be helpful if the authors discussed why they see these differences in a bit more detail, especially with regard to what the data imply about whether PrPC glycosylation or PrPSc glycosylation influences the phenotype of BSE PrPSc deposition in vivo.

We have included a more detailed description of the neuropathology in TgBo110 mice. To our knowledge, the neuropathological characteristics of humanized and bovinized models when inoculated with BSE isolates are, although similar, not identical. Florid plaques are a characteristic neuropathological feature of BSE prions when transmitted to humans/humanized models (Ironside JW, Head MW. 2004;284:133-59; Will RG, Ironside JW, et al. Lancet. 1996;347(9006):921-5), whereas in TgBo110 mice inoculated with BSE, punctate, granular and plaque-like deposits in the neuropil are observed (Castilla J, Adan AG, et al. Arch Virol. 2003;148(4):677-91; Huor A, Espinosa JC,et al. Proc Natl Acad Sci U S A. 2019; 16;116(52):26853–62).

The distribution of lesions displayed by TgNN6h and TgBo110 mice in our study is also very similar to that described by other authors. As previously reported, neuropathological lesions in humanized mice inoculated with vCJD mainly involve thalamic, hippocampal and cerebral cortex areas (Padilla D, Beringue V, et al. PLoS pathogens. 2011;7(3):e1001319), very similar to those observed by us in TgNN6h mice. On the other hand, most affected brain areas in TgBo110 inoculated with BSE are medulla, thalamus and septal nuclei, with low scores in cerebellar cortex and hippocampus (Huor A, Espinosa JC,et al. Proc Natl Acad Sci U S A. 2019; 16;116(52):26853–62; Douet, JY., Huor, A., et al. Acta Neuropathol.2021; 141, 383–397), results that also coincide with our lesional patterns in TgBo110 mice.

The manuscript has been modified according to your suggestions:

Lanes 277-283 (results):

In addition, these mice showed widespread spongiform degeneration, especially prominent in septal area, thalamus, medulla oblongata and pons, with lower scores in hippocampus, mesencephalon, and cerebellar cortex. This distribution of neuropathological lesions is very similar to that observed by other authors in BoTg110 mice inoculated with BSE (43, 44). All BoTg110 mice that succumbed to disease developed fine-punctate, granular and plaque-like deposits, following a similar brain distribution (Figure 6). This morphological pattern has been described before in the same model inoculated with BSE isolates (4, 43).

Lane 393-397 (discussion):

In fact, neuropathological features in BoTg110 mice inoculated with the TgNN6h-passaged BSE prions were very similar from those reported by us and other authors in BoTg110 mice inoculated with fully glycosylated BSE prions, regarding distribution (43, 44) and morphology of PrP^Sc^ deposits (4, 43).

**Reviewer #3:** Otero et al. describe results that vCJD, BSE & BSE passaged through sheep and porcine can seed formation of prions with an unglycosylated human PrP substrate in PMCA, that subsequently can be passaged back into BoTg110 mice with restoration of BSE phenotype. They also demonstrate biochemical evidence that the protease resistant core and infectivity is maintained with in vitro PMCA propagation to suggest glycosylation is not needed to maintain strain properties with passaging. Strong support to indicate lack of glycosylation does not ultimately impede BSE-initiated conversion of human PrP that maintains overall BSE strain properties is provided with evidence that the generated prions can propagate in BoTg110 mice.

This study provides interesting and further evidence that “strainness” is dictated by the structural backbone of a prion strain, and not fundamentally altered by glycans overall. However, and considering the author’s own data that passaged unglycosylated BSE-seeded prions result in incomplete attack rates in BoTg110 mice, it is this reviewer’s perspective that strong statements such as that glycans do not alter the “strength” of the transmission barrier or are “not relevant” in maintenance of strain properties is not fully indicated by the data here that cannot account for potential subtleties in propagation kinetics or conformation/structures as it relates to glycosylated and unglycosylated forms. Such strong statements should be avoided or rephrased.

1) Does a 1% compared to 10% brain homogenate (10-fold difference) sufficiently reconcile the discrepancy between the results reported here in BoTg110 mice, when compared to those previously reported? Authors should discuss titers, and if a 10-fold decrease based solely on titer would be expected to result in lack of disease in these models, or if this is speculative.

2) The authors reason that the incomplete attack rate above is likely attributable to 1) a 0.6x expression level of the TgNN6h mice that ultimately results in less PrPSc accumulation for subsequent passage and 2) Use of 10-fold less material (see point 1). However, these statements rely on the assumption that less PrPSc eventually accumulates that, per amount brain tissue, would be available for subsequent passage. Evidence should be provided/discussed that less PrPSc is known to ultimately accumulate in the TgNN6h mice when compared to WT.

The reviewer is right in pointing that the differences between our results and those previously reported should be discussed in terms of titers. The discussion includes three potential reasons for the incomplete attack rates on this model with TgNN6h passaged inocula, including the existence of a transmission barrier between human and cattle (further enhanced due to N to Q substitutions in TgNN6h models), which may be higher than the barrier between sheep and cattle, as in the transmission studies previously reported. Nonetheless, we agree that this difference needs to be further explained and that hypothesizing about titer differences due to inoculum preparation (1 % vs 10 %), and lower PrP^C^ expression levels of the TgNN6h model may not be sufficient. For that reason, and in addressing the comments from another reviewer, we decided to perform an *in vitro* titration experiment, in order to demonstrate lower infectivity in TgNN6h-passaged inocula than in the original cattle-BSE isolate. We used PMCA to compare the propagation of the cattle BSE isolate with that of the BSE, sBSE, and pBSE-inoculated TgNN6 brain homogenates that were used to inoculate BoTg110 mice. As explained in the now revised version of the Materials and Methods section, the four brain homogenates were serially diluted (10^-1^ to 10^-6^) and used as seeds on PMCA using a substrate prepared from healthy BoTg110 mice. All the samples were subjected to two serial 24 h rounds of PMCA in the same conditions as previous PMCA reactions, and performing a 1:10 dilution in between the two serial rounds. PMCA products from the second round were analyzed by Western blot, which is shown now as Supplementary figure 4. As shown in the gel, the original cattle BSE isolate was detectable up to dilution 10^-5^, whereas the three TgNN6h brain homogenates were only able to propagate up to dilution 10^-1^, i.e. a 10,000-fold lower *in vitro* propagation capability. This lower titer, together with the prolonged incubation times characteristic of BSE isolates, could explain the incomplete attack rates observed.

As replied above according to another reviewer’s suggesting, the Results section now includes a paragraph explaining the results of this new experiment (lanes 245-262). Also, the Discussion section has been modified to contemplate all possible explanations described above and to include a reference to Supplementary figure 4 that shows the results of the *in vitro* titration experiment (lanes 383-407). Finally, a paragraph has been introduced in the Material and Methods section explaining the technical details of this new experiment (lanes 490-495).

3) Why were different PK conditions used with TgNN6h propagated materials (Fig 2)? If different PK conditions were required for efficient digestion, does this argue against complete maintenance of all strain properties? For direct comparison, the same PK concentrations should be used. The resolution to a single band for the TgNN6h substrate is interpreted to indicate that BSE, sBSE, pBSE and vCJD yield the same product. However, sBSE and vCJD seeded PMCA prions appear less protease resistant (less material following digestion on blot) which could indicate differences in assembly and/or conformation. Digestion at different PK amounts, including a limited PK digestion, or another approach to assess conformation of the BSE, sBSE, pBSE, and vCJD PMCA products would better indicate if conformational differences, albeit ultimately not prohibitive towards propagation in the animal models, do exist and contribute to differences in PK resistance observed.

PMCA-propagated isolates shown in Figure 2 were obtained after serial PMCA passaging in three different substrates, with distinct PrP^C^ sequences and expression levels, as well as different microenvironments. TgNN6h substrate was prepared from perfused mouse brains that were immediately frozen after extraction and handled under laboratory conditions at all times, while cattle and, specially, human substrates were prepared from brain samples whose handling was largely unknown to us but that most likely were not as careful as that of TgNN6h brains. Therefore, we believe that the need to use different concentrations of PK to achieve correct digestion of the three types of isolates is due to these differences in substrate intrinsic characteristics and qualities, and not to actual strain variability.

We have now included a paragraph in the Material and Methods section (lanes 519-521) specifying the reasons why we used different PK concentrations:

PK concentration was empirically determined for each type of isolate in order to achieve complete digestion in all cases, due to the differences in PrP^C^ sequence, expression levels and microenvironment.

In any case, the aim of Figure 2 is not to compare the relative PK resistance of the isolates, but rather to verify that there are no noticeable changes in the banding patterns after PMCA passaging. In relation to this, we would like to point out that careful comparisons of PK resistance between prion isolates are normally done with PK concentrations differing by 10/50-fold (and not 2-fold as is the case here between 170 and 85 μg/mL).

Regarding the difference in signal intensity between BSE/pBSE and sBSE/vCJD after PMCA in TgNN6h, this is due to the fact that these PMCA products were analyzed directly by SDS-PAGE and Western blot, without previous adjustment of the quantities loaded in the gel. Given that PMCA is a highly stochastic technique, the efficiency among serial rounds is variable, and thus the distinct signals observed are likely due to dissimilarities in PrPSc amount rather than to different resistances to PK.

A paragraph has been included in the Results section (lanes 125-131) clarifying this:

As expected, given the absence of different glycoforms in TgNN6h PrP^C^, PMCA products derived from the propagation of all four BSE sources on TgNN6h substrate showed a PrP^Sc^ signature characterized by a single, non-glycosylated band at 19 kDa (Figure 2). Given that PMCA is a highly stochastic technique (the efficiency among rounds can differ significantly), and that these samples were submitted for SDS-PAGE and Western blot without prior adjustment of quantities, the differences in signal intensity observed among different TgNN6h-propagated isolates is likely due to dissimilarities in PrP^Sc^ amount rather than to different resistances to PK.

*Minor:*

Typo on line 387 “on a on a”

Thank you, the typo has been corrected.

Sincerely,

Dr. Castilla

IKERBasque Research Professor
CIC bioGUNE
